# PD-L1/PD-L2-expressing B-1 cells inhibit alloreactive T cells in mice

**DOI:** 10.1371/journal.pone.0178765

**Published:** 2017-06-01

**Authors:** Takayuki Hirose, Yuka Tanaka, Asuka Tanaka, Hiroshi Sakai, Yu Sasaki, Nobuo Shinohara, Hideki Ohdan

**Affiliations:** 1 Department of Gastroenterological and Transplant Surgery, Applied Life Sciences, Institute of Biomedical & Health Sciences, Hiroshima University, Hiroshima, Japan; 2 Department of Renal and Genitourinary Surgery, Graduate School of Medicine, Hokkaido University, Sapporo, Japan; Beth Israel Deaconess Medical Center, UNITED STATES

## Abstract

B cells constitute a complex system of antigen-presenting cells (APCs) and exist as distinct subsets that differ in their lineage affiliation, surface molecule expression, and biological function, thus potentially regulating the immune response. In this study, we investigated the immune-regulatory roles of murine B cell subsets as regulatory APCs targeting alloreactive T cells. Either splenic B cells, peritoneal cavity (PerC) B cells, or non-B cells from Balb/c mice were intravenously injected into B6 mice. Serum levels of anti-Balb/c antibodies in the recipients of PerC B cells were significantly lower than those in the recipients of splenic B cells and PerC non-B cells, as determined over a 4-week period after the injection. Mixed-lymphocyte reaction (MLR) assays using splenocytes from the B6 mice at 2 weeks after the injection revealed the significantly reduced anti-Balb/c T cell-responses in the recipients of PerC B cells, as compared to those in the recipients of splenic B cells or untreated control mice. Since PerC B cells contained MHC class II^+^ CD80^+^ CD86^+^ PD-L1^+^ PD-L2^+^ cells among the CD5^+^ B-1a cell subset, PerC B cells from Balb/c mice were pre-incubated with anti-PD-L1/PD-L2 mAbs prior to injection. This treatment abrogated their immune-regulatory effects on anti-Balb/c T cells in the MLR assays. In addition, the inoculation with Balb/c PerC B cells significantly prolonged the survival of subsequently grafted Balb/c hearts in B6 mouse recipients, whereas that with SPL B cells did not. These findings indicate that the PerC B cells, including PD-L1/PD-L2 B-1a cells, may suppress T cells responding to allostimulation, and thus may be optimal for donor lymphocyte injection.

## Introduction

T-cell activation during sensitization or rejection in organ transplant recipients first needs antigen presentation to T cells by antigen-presenting cells (APCs), such as macrophages, dendritic cells, and B cells. B cells are well known to play a role in allostimulation of T cells [1–4]. B cells not only constitute a complex system of APCs but also exist as distinct subsets that differ in their lineage affiliation, surface molecule expression, and biological function, thus potentially regulating the immune responses. B cells play a central role in humoral immunity, but specific B cell subsets can also regulate T cell responses to foreign and self-antigens. These B cells are called regulatory B cells (Breg), and different types have been reported to date [5–16].

As one part of Breg cells, B cells were found to express programmed death-ligand (PD-L) 1 and PD-L2, which are one of the key factors responsible for inhibitory T cell signaling, providing immune homeostasis and mediating the mechanisms of tolerance [17–20], PD-L1 is constitutively expressed by various immune cells and nonimmune cells, whereas PD-L2 expression is expressed only by APCs such as macrophages and dendritic cells [21]. In addition, constitutive expression of PD-L2 together with PD-L1 is observed on 50–70% of mouse peritoneal CD5^+^ B-1a cells, a unique B cell subset, that act as efficient APCs [17]. Although the molecular and cellular functions of PD-L1 and PD-L2 in these B-1a cells currently remain unclear, these molecules per se are known to have a physiological role in regulating autologous T cell-immune responses in self-immunity by engaging PD-1.

This knowledge prompted us to investigate if the unique B cell subset that expresses PD-L1 and PD-L2 also inhibits alloimmune T cell responses. The present study has demonstrates that the infusion of allogeneic PD-L1^+^/PD-L2^+^ B-1 cells suppresses T cells responding to cognate allostimulation. Thus, the present study provides a novel approach to induce T-cell hyporesponsiveness against transplanted allografts by infusing donor specific B-1 cells.

## Materials and methods

### Mice

C57BL/6 (B6; H-2D^b^) and Balb/c (H-2D^d^) mice were purchased from CLEA Japan (Tokyo, Japan). All the mice were housed in the animal facility of Hiroshima University, in a pathogen-free, micro-isolated environment and were used at an age of 8–12 weeks. All experiments were approved by the Institutional Review Board of Hiroshima University and were handled and conducted according to the guidelines of the National Institutes of Health (publication no.86-23, revised 1996). Animal welfare was carefully ensured constantly by experienced operators every day. Mice were euthanized by cervical dislocation after inhalation of isofluran, when indicated. All efforts were made to minimize suffering of the animals along all the duration of their life and during the sacrifice.

### Cell preparation

Mononuclear cell suspensions from the spleen (SPL), peritoneal cavity (PerC), liver, bone marrow (BM), cervical lymph node (CLN), and peripheral blood (PB) were prepared. B cells from the mouse SPL or PerC were purified by performing positive selection with CD19 MicroBeads (Miltenyi Biotec, San Diego, CA, USA) and an autoMACS Separator (Miltenyi Biotec), according to the manufacturer’s instructions. Non-B cells from the PerC were purified by negative selection at the same time. Purity of the isolated B and non-B cells was assessed by flow cytometry (FCM) and was typically found to be >95%.

When indicated, 10 × 10^6^ of B cells isolated from PerC of Balb/c mice were incubated with anti-PD-L1 (10F.9G2, BioLegend, San Diego, CA, USA) and/or anti-PD-L2 (TY25, BD Pharmingen, San Jose, CA, USA) monoclonal antibodies (mAbs) for 30 min at 37°C in a 5% CO_2_ incubator prior to injection. Rat immunoglobulin G (IgG) 2b (R35-38, BD Pharmingen) and IgG2a (R35-95, BD Pharmingen) were used as isotype-matched control IgGs for anti-PD-L1 and anti-PD-L2 mAbs, respectively. All cell cultures were performed in RPMI 1640 medium supplemented with 5% FBS, 2 mM L-glutamine, 100 U/ml penicillin-streptomycin, and 50 μM 2-ME.

### FCM analysis

Mononuclear cells freshly isolated from the SPL, PerC, liver, BM, CLN, and PB were incubated for 30 min at 4°C with the following diluted fluorescently labeled mAbs: PD-L1-phycoerythrin (PE) (MIH5; BD Pharmingen), PD-L2-PE (TY25; BD Pharmingen), FasL-PE (MFL3; BD Pharmingen), CD80-PE (16-10AI; BD Pharmingen), CD86-PE (GL1; BD Pharmingen), CD4-PE (L3T4; BD Pharmingen), CD5-PE-Cy7 (53–7.3; BioLegend), CD8a-PE (53–6.7; BD Pharmingen), CD8a-PE-Cy7 (53–6.7; BD Pharmingen), CD11b-APC-Cy7 (M1/70; BD Pharmingen), CD19-FITC (1D3; BD Pharmingen), IgM-allophycocyanine (APC) (II/41; BD Pharmingen), H-2D^d^-PE (34-2-12; BD Pharmingen), CD40-PE (3/23; BD Pharmingen), TRAIL-PE(N2B2; eBioscience, San Diego, CA, USA), and IA/IE-PE(M5/114.15.2 BD Pharmingen). The following isotype matched control IgGs were used; mouse IgG 2a,k-PE, Hamster IgG1,λ1-PE-Cy7, mouse IgG2a,k-FITC, mouse IgG1-APC, and mouse IgG1-APC-Cy7. Mixed lymphocyte reaction (MLR) assays were performed using an intracellular fluorescent dye carboxyfluorescein diacetate succinimidyl ester (CFSE) (CFSE-MLR) were performed. FCM assays were performed on either a FACSCalibur (BD Biosciences) or FACSCanto II (BD Biosciences). Nonspecific FcγR binding of labeled mAbs was blocked using anti CD16/32 mAb (2.4G2; BD Pharmingen). Dead cells were excluded from the analysis by forward scatter and propidium iodide (PI; Sigma-Aldrich, St. Louis, MO, USA) or 7-AAD (BD Pharmingen).

### Adoptive B cell transfer

B6 recipient mice received a total of 10 × 10^6^ of Balb/c B cells isolated from SPL or PerC, or non-B cells from PerC in a volume of 200 μL via tail vein injection. Splenic T cell response was analyzed 14 days after the adoptive transfer by performing CFSE-MLR assay as described below. To assess the allo-Ab response in recipient mice, blood samples of recipient mice were obtained before injecting the cells and weekly thereafter for 4 weeks. Serum was isolated and was frozen for allo-Ab analysis by FCM assay.

### Assay of allo-Ab

Anti-Balb/c Abs were detected by indirect immunofluorescence staining of Balb/c splenocytes by FCM assay. A total of 0.5 x 10^6^ splenocytes were incubated with 100 μL of serum for 1 h at 4°C, followed by incubation for 30 min at 4°C with FITC-conjugated rat anti-mouse IgM mAb (R6-60.2; BD Pharmingen) or FITC-conjugated anti-mouse IgG polyclonal Ab (Poly4060; BioLegend), and PE-conjugated anti-mouse CD19 (1D3; BD Pharmingen) were used to detect production of allo-IgM or IgG Abs. Similarly, biotin-conjugated rat anti-mouse IgG1 mAb (A85-1; BD Pharmingen), rat anti-mouse IgG2a/2b mAb (R2-40; BD Pharmingen) or rat anti-mouse IgG3 (R40-82; BD Pharmingen), and PE-conjugated anti-mouse CD19 were used to detect production of allo-Ab (IgG) subclasses. Median fluorescence intensity (MFI) of CD19-negative cells was used to determine donor-specific Ab levels.

### CFSE-MLR assay

Splenocytes of B6 recipients at 2 weeks after adoptive transfer were labeled with 5 μM CFSE (Molecular Probes: Eugene, OR) as described previously [22,23], and were resuspended in culture medium for use as responder cells. Fractions of whole splenocytes were prepared from B6 mice that received SPL B cells, PerC B cells, or PerC non-B cells. After each fraction had been irradiated (30 Gy) for use as stimulator cells, 4 × 10^6^ stimulator cells and 4 × 10^6^ responder cells were cocultured in 24-well, flat-bottom plates at 37°C in a 5% CO_2_ incubator in the dark. After 4 days of incubation, the responder cells were harvested and analyzed by FCM. CD4^+^ and CD8^+^ T cells were selected through gating and analyzed for intensity of CFSE fluorescence. Stimulation indexes (SI) and precursor frequencies (PF) were calculated as described previously [22].

### Heart transplantation

Two weeks before heart transplantation, recipient B6 mice were intravenously injected with 10 × 10^6^ SPL or PerC CD19^+^ B cells from Balb/c mice. Prior to surgery, mice were anesthetized by intraperitoneal injection of xylazine (5 mg/kg body weight) and ketamine (100 mg/kg body weight). Cervical heterotopic heart transplantation from Balb/c mice into naïve B6 mice or B cell injected B6 mice was performed using a modified cuff technique [24]. Briefly, the right external jugular vein and the right common carotid artery were dissected free and were fixed to the appropriate cuffs composed of polyethylene tubes. For anastomoses, the aorta and the main pulmonary artery of the obtained donor heart were drawn over the external jugular vein, respectively. Graft ischemic time for the transplanted hearts was < 30 min. The function of the grafts was monitored daily through inspection and palpation. Rejection was determined based on the cessation of heart beat and was confirmed by performing histological analysis. Mice were daily monitored for weight and well being. If there was evidence of more than 15% weight loss compared to weight at surgery-date, no food intake, breathing difficulties, apathy, or a hunched position as well as when the heart was rejected, animals were euthanized. Two mice were excluded from the evaluation of graft survival because they suddenly died of unknown cause with beating allograft heart.

### Statistical analysis

Data are presented as mean ± standard error of mean (SEM). Statistical analysis was performed using Student’s *t* test (GraphPad Prism, San Diego, USA). Survival of the heart allografts was calculated using Kaplan-Meier/log-rank test. A p-value of < 0.05 was considered statistically significant.

## Results

### Naïve B-1a cells from PerC highly expressed PD-L1, PD-L2, CD80, CD86, and MHC class II

**Fig 1:**
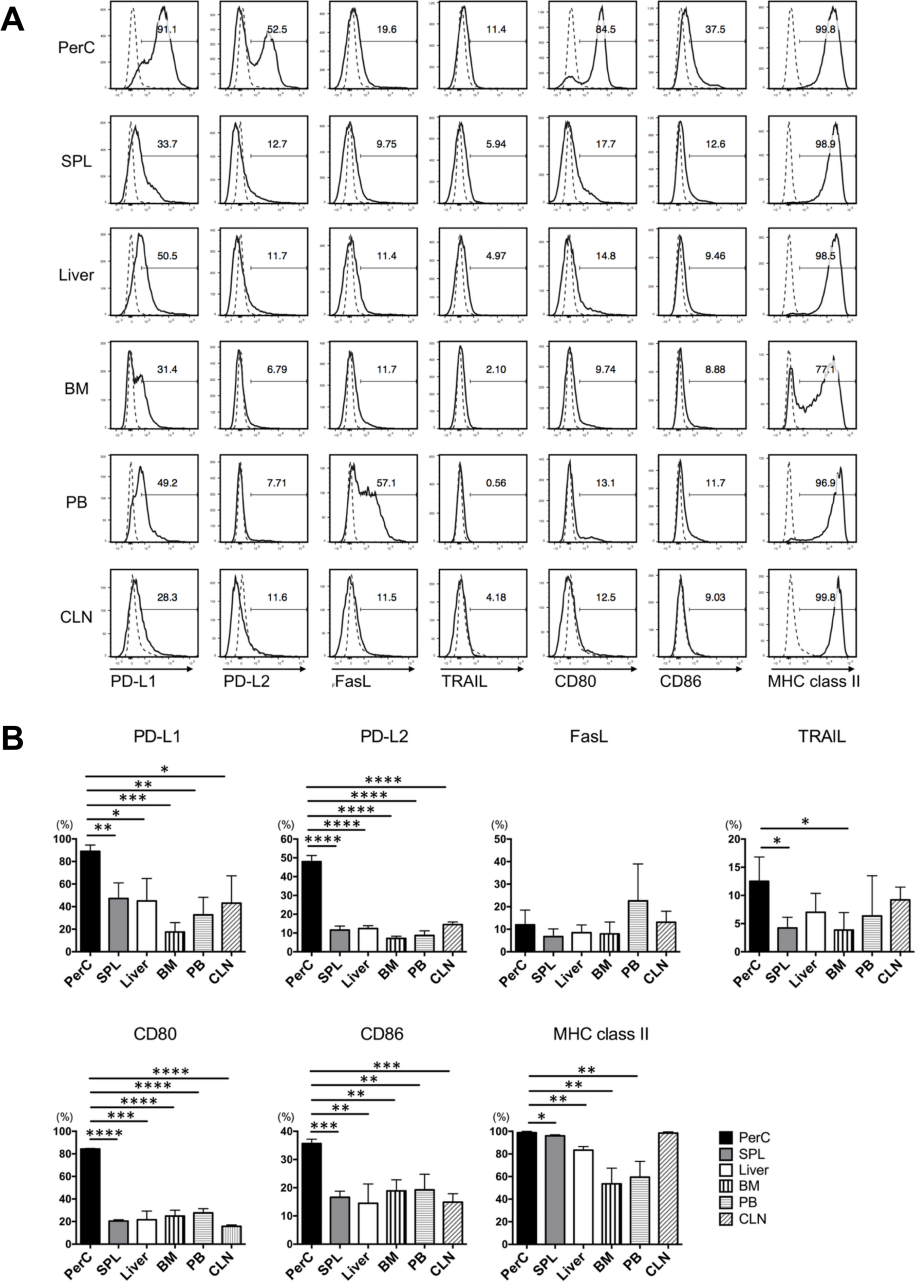
Phenotypic analysis of B cells in the organs of Balb/c mice. We investigated the phenotypic characteristics of naïve B cells isolated from the PerC, SPL, liver, BM, PB, and CLN of Balb/c mice by performing anatomical screening of immune-regulatory markers PD-L1, PD-L2, FasL, and TRAIL and co-stimulatory membrane markers CD80, CD86, and I-A/I-E (MHC class II) by performing FCM analysis. Anti-CD19 mAb was used as a B cell marker. (A) Histograms indicating representative FCM results of phenotypic analysis of B cells. Dashed lines indicate the isotype-matched control IgG. (B) Percentage (mean ± SEM) expression of each membrane marker expressed on B cells from each organ. *p < 0.05, **p < 0.01, ***p < 0.001, and ****p < 0.00001 (Student’s *t*-test). Data are representative of three experiments with three mice per group.

To search for B cells having the potential to induce hyporesponsiveness of T cells to allostimulation, we investigated the phenotypic characteristics of naïve B cells from SPL, PerC, liver, BM, PB, and CLN in an anatomical screening for immune-regulatory and co-stimulatory membrane markers. Expressions of MHC class II, CD80, and CD86—surface molecules necessary for efficient antigen presentation to T cells—and the immune-regulatory molecules anti-PD-L1, PD-L2, FasL, and TRAIL were analyzed. In Balb/c mice, naïve B cells from PerC showed remarkably higher levels of PD-L1, PD-L2, CD80, and CD86 compared to those in other organs, while FasL and TRAIL were rarely expressed on B cells from any organs (Fig 1A). Mean percentage of PD-L1, PD-L2, CD80, and CD86 expression on B cells from PerC was statistically higher than that from any other organ (Fig 1B. FasL and TRAIL on B cells from each organ showed generally low frequency and no difference was detected in all 6 organs. MHC class II was expressed on almost all B cells. Similar results were obtained for B cells isolated from B6 mice (S1 Fig).

We further analyzed the expression of immune-regulatory molecules on the various B cell subsets isolated from the SPL, PerC, and liver. SPL cells were stained with combinations of mAbs directed against IgM and CD21 with a variety of immune-regulatory molecules. Since marginal zone (MZ), follicular (FO), and newly formed B cells, which constitute anatomically distinct B cell subsets in the SPL, differ in their expression of IgM and CD21 [25], a combination of these markers can be used to select these three subsets by gating (S2 Fig). CD21^high^ IgM^high^ MZ B cells, which are uniquely positioned near antigen trapping cells in the marginal sinus, where their association with macrophages facilitates their exposure to blood-borne antigens [25,26], exclusively expressed a high level of PD -L1, but barely expressed PD-L2. The expression of those PD-L1/L2 was limited on CD21^int^ IgM^int^ FO B cells, which were positioned adjacent to T cell areas, facilitating their response to T cell-dependent antigens and promoting germinal center formation [26], and CD21^-/low^ IgM^high^ newly formed B-0 cells, which had recently immigrated from the BM into the SPL.

**Fig 2:**
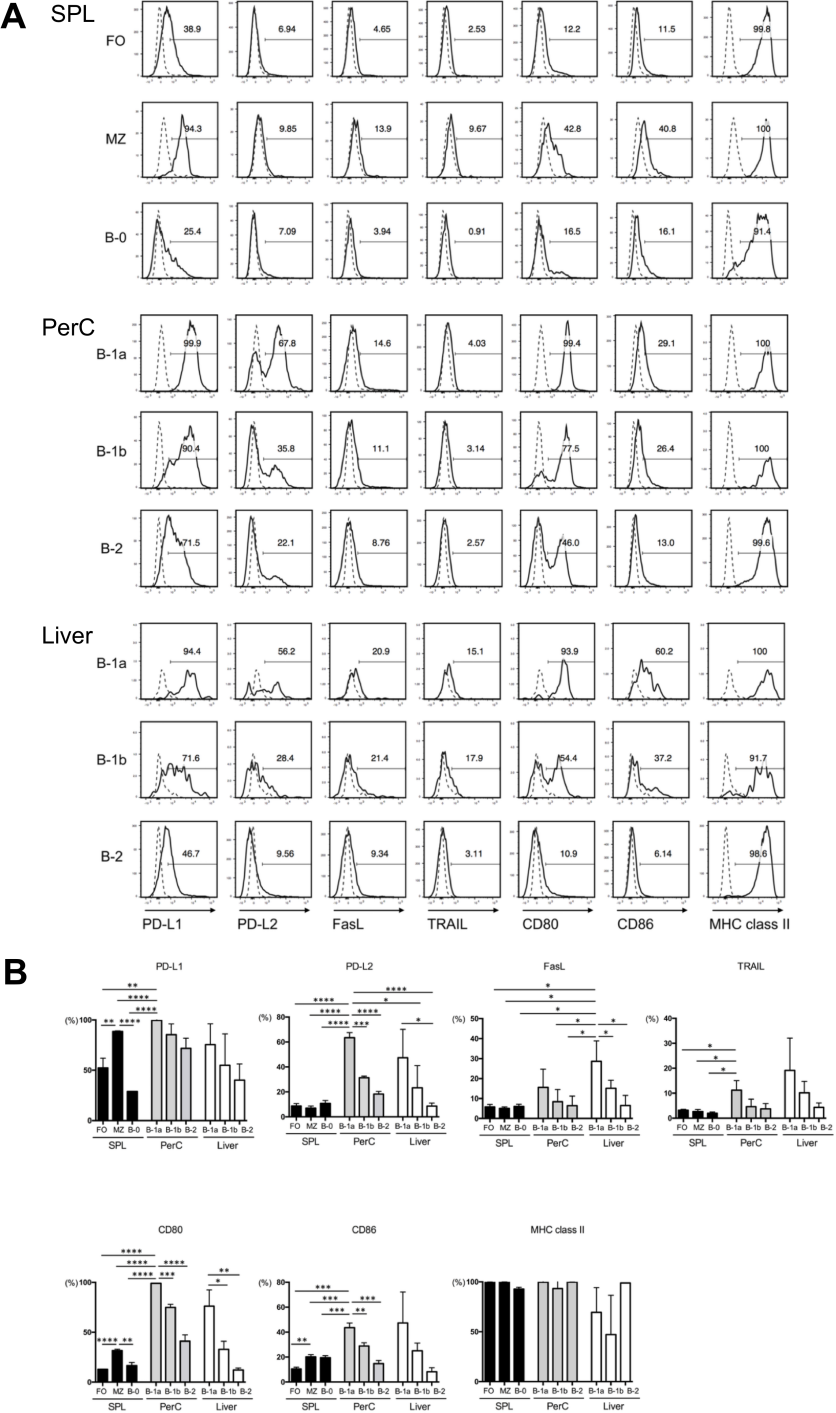
Phenotypic analysis of each B cell subset isolated from Balb/c mice. Naïve B cells from the SPL, PerC, and liver were stained with various combinations of mAbs directed against IgM, CD19, CD21, CD11b, CD5, PD-L1, PD-L2, FasL, TRAIL, CD80, CD86, and MHC class II, and were analyzed by performing FCM. Then, these cells were divided into the following subsets: CD21^int^IgM^int^ follicular (FO) B cells, CD21^high^IgM^high^ marginal zone (MZ) B cells, and CD21^-/low^IgM^high^ B-0 cells from the SPL, and CD11b^+^CD5^+^ B-1a cells, CD11b^+^CD5^-^ B-1b, and CD11b^-^CD5^-^ B-2 cells from the PerC and liver (S2 Fig). (A) Histograms of PD-L1, PD-L2, FasL, TRAIL, CD80, CD86, and MHC class II on each B cell subset by performing FCM analysis. (B) Percentage (mean ± SEM) expression of each membrane marker expressed on each B cell subset. *p < 0.05, **p < 0.01, ***p < 0.001, and ****p < 0.00001(Student’s *t*-test). Data are representative of three experiments with three mice per group.

PerC and liver B cells are divided into three distinct subsets, CD11b^+^CD5^+^ B-1a cells, CD11b^+^CD5^-^ B-1b, and CD11b^-^CD5^-^ B-2 cells (S2 Fig). Distinct from the conventional subset B-2 cells, the role of B-1a and B-1b cells as effectors of innate-like immunity is widely accepted, although the functional difference between B-1a and B-1b cells remains to be resolved [19,27–36]. B-1a cells from PerC and liver demonstrated high frequency of PD-L1, PD-L2, CD80, and CD86 expression compared to those from the other subsets (Fig 2A). Mean percent expression of PD-L1, PD-L2, CD80, and CD86 was significantly higher in PerC B-1a cells than any other subsets of B cells from SPL, PerC, and liver (Fig 2B). These B cells expressed surface molecules necessary for the efficient Ag-presentation to T cells, as well as apoptosis-inducing ligands, potentially imparting their tolerogenic potential upon the alloantigen recognition of T cells.

### Intravenous injection of allogeneic PerC B cells prevented sensitization

**Fig 3:**
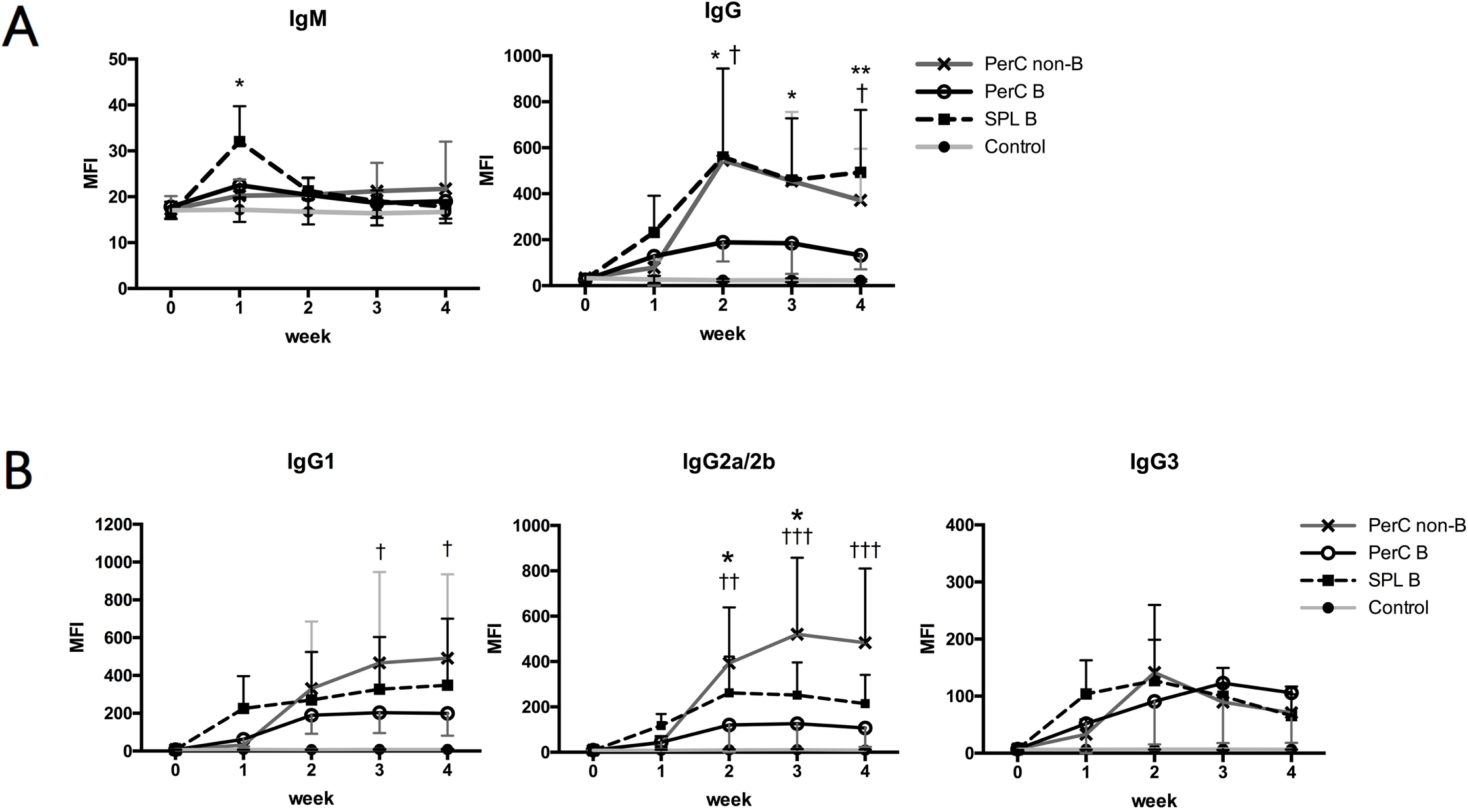
Allo-Ab assay after intravenous injection of allogeneic B cells. B cells of SPL or PerC, or non-B cells of PerC isolated from Balb/c mice were injected into B6 mice trough the tail vein. To evaluate allo-Ab production, sera of recipient B6 mice were obtained every week after the adoptive transfer, and were incubated together with splenocytes from Balb/c mice as target cells. Mice in the control group received no adoptive transfer of cells. (A) Allo antibody response after the intravenous injection. Anti-Balb/c IgM or IgG was evaluated by FCM assay, and MFI was calculated (error bars, SEM). CD19^+^ B cells of the Balb/c splenocytes were excluded from the analyses, because they included surface IgM or IgG expressing cells. (B) Subclasses of anti-Balb/c IgGs produced after the intravenous injection. Anti-IgG1, anti-IgG2a/2b, or anti-IgG3 was used to detect the production of allo-IgG subclasses and evaluated by FCM assay. Data are presented as MFI ± SEM; *p < 0.05, and **p < 0.01 (mice receiving PerC B cells vs mice receiving SPL B cells); †*p* < 0.05, ††, and *p* < 0.01, and ††† *p* < 0.001 (mice receiving PerC B cells vs mice receiving PerC non-B cells). Data are representative of two independent experiments, with six mice per group.

To investigate the immunogenicity of SPL and PerC B cells, 10 × 10^6^ B cells from SPL, PerC, or non-B cells isolated from PerC of naïve Balb/c mice were injected to B6 mice through the tail vein and production of anti-Balb/c Abs in the sera of recipient B6 mice was serially evaluated. In B6 mice receiving SPL B cells, serum levels of anti-Balb/c IgM were transiently elevated 1 week after the inoculation and declined thereafter (Fig 3A; left). However, the sera of B6 mice receiving PerC B or non-B cells did not show similar increase in anti-Balb/c IgM levels. In B6 mice receiving SPL B cells or PerC non-B cells, serum levels of anti-Balb/c IgG increased over time and reached a plateau 2 weeks after the inoculation (Fig 3A; right). However, this increase in anti-Balb/c IgG level was not observed in B6 mice receiving PerC B cells. Subclass analyses of anti-Balb/c IgGs showed that IgG2a/b was predominant in the recipients of PerC non-B cells, whereas no particular uneven class-switching was observed in recipients of SPL or PerC B cells (Fig 3B). Thus, intravenous injection of allogeneic PerC B cells prevents significant sensitization.

### Intravenous injection of allogeneic PerC B cells inhibited T cell-immune responses to cognate allostimulation

**Fig 4:**
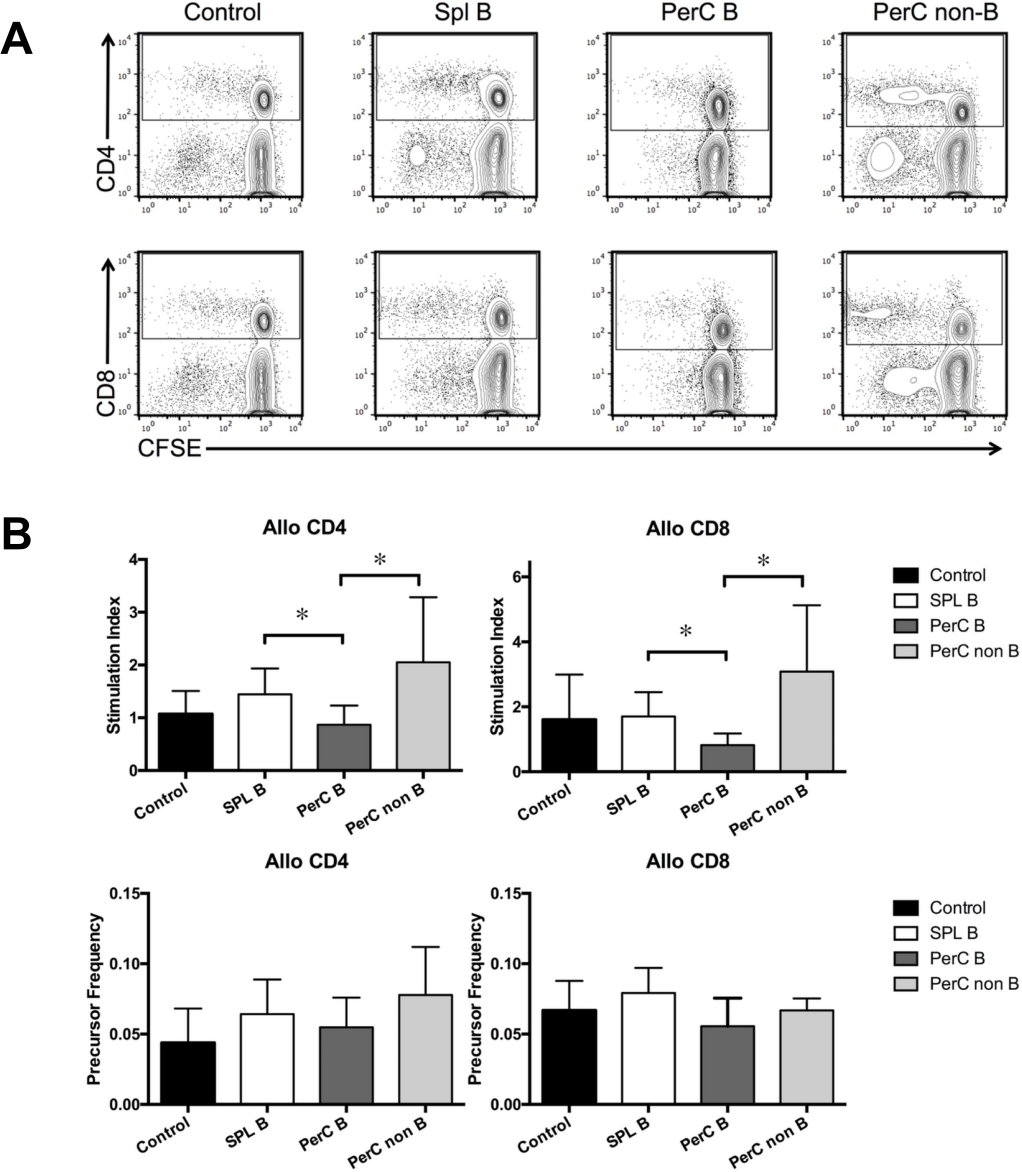
T-cell allo-response after the intravenous injection of allogeneic B cells, as evaluated by performing CFSE-MLR assay. SPL or PerC B cells, or PerC non-B cells from Balb/c mice were isolated by magnetic sorting with CD19 microbeads, and were injected into B6 mice through the tail vein. T-cell alloreactivity was determined by performing CFSE-MLR assay 2 weeks after the adoptive transfer. The CFSE-MLR assay was performed using B6 mice injected with Balb/c SPL B cells (n = 6), Balb/c PerC B cells (n = 6), and Balb/c PerC non-B cells (n = 6); control B6 mice were not injected with any cells (n = 7), as responders. SIs and PFs were calculated as described previously [22]. (A) CFSE-labeled CD4^+^ (upper row) and CD8^+^ (lower row) T cell division in the MLR assay was quantified by performing FCM analysis. Representative plots and images are shown. CFSE-labeled splenocytes from B6 mice were used as responders, and irradiated (30 Gy) splenocytes from Balb/c mice were used as stimulators. (B) The SIs and PFs of splenocytes from B6 mice in the CFSE-MLR are shown. *p < 0.05. Data are presented as mean ± SEM, and are representative of two independent experiments with six to seven mice per group.

Next, we investigated the immune-regulatory effects of SPL and PerC B cells on allogeneic T cells by performing the CFSE-MLR assays. Two weeks after the injection, splenocytes of B6 mice were harvested and assayed by performing the CFSE-MLR. The SI of anti-Balb/c CD4^+^ T cells was significantly lower in mice receiving PerC B cells than in mice receiving SPL B cells or PerC non-B cells (*p* < 0.05 for mice receiving PerC B cells vs mice receiving SPL B cells and mice receiving PerC B cells vs mice receiving PerC non-B cells). The SI of anti-Balb/c CD8^+^ T cells was also significantly lower in mice receiving PerC B cells than in mice receiving SPL B cells and PerC non-B cells (*p* < 0.05 for mice receiving PerC B cells vs mice receiving SPL B cells and mice receiving PerC B cells vs mice receiving PerC non-B cells). Moreover, the PF of anti-Balb/c CD4^+^ or CD8^+^ T cells was lower in mice receiving PerC B cells than in mice receiving SPL B cells or PerC non-B cells, however, the difference in PFs between mice receiving PerC B cells and those receiving SPL B cells or PerC non-B cella was not statistically significant (Fig 4). These results indicate that the inoculation of allogeneic PerC B cells inhibits T cell-immune responses to the cognate allostimulation.

### PerC B cells pre-incubated with blocking anti-PD-L1/PD-L2 mAbs abrogated immune-regulatory effects on alloreactive T cells

**Fig 5:**
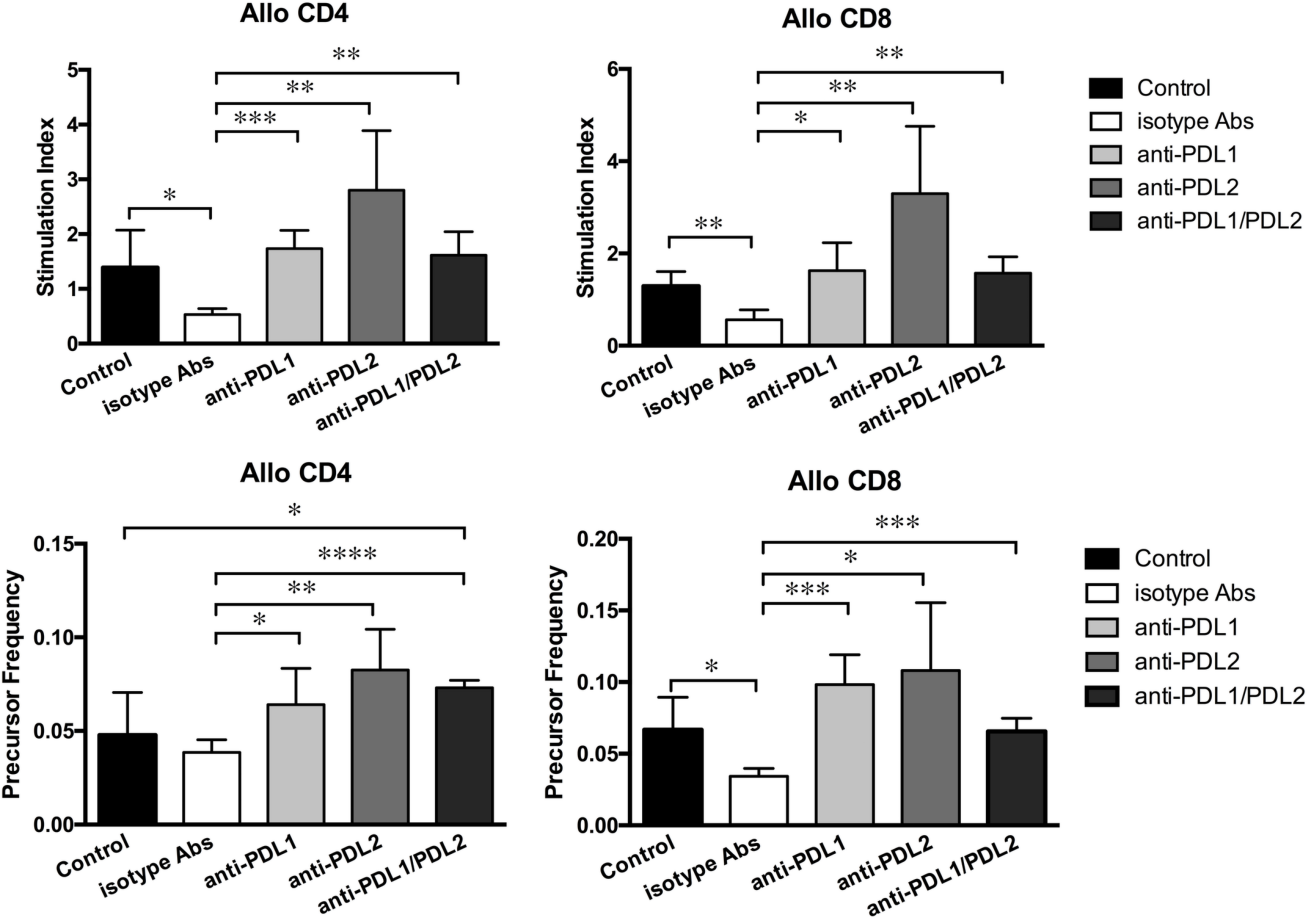
CFSE-MLR assay after the intravenous injection of PerC B cells pre-incubated with anti-PD-L1 and/or anti-PD-L2 mAbs. PerC B cells from Balb/c mice were isolated, pre-incubated with anti-PD-L1 and/or anti-PD-L2 mAb for 30 min, and injected to B6 mice through the tail vein. Isotype-matched control IgGs were used instead of anti-PD-L1 or anti-PD-L2 mAb. Rat IgG2b was used instead of anti-PD-L1 mAb, and Rat IgG2a was used instead of anti-PD-L2 mAb. Mice in the control group did not receive adoptive transfer of cells or mAbs. T-cell alloreactivity was determined by performing the CFSE-MLR assay 2 weeks after the intravenous injection. Upper row shows the mean SIs of CD4^+^ and CD8^+^ T cells, and lower row shows the mean PFs of CD4^+^ and CD8^+^ T cells; *p < 0.05, **p < 0.01, ***p < 0.001, and ****p < 0.00001. Data are presented as mean ± SEM, and are representative of two independent experiments with four to five mice per group.

To address the functional significance of PD-L1 and PD-L2 expression on PerC B cells in the hyporesponsiveness of alloreactive T cells, we performed the CFSE-MLR assay by using the splenocytes isolated from B6 mice injected with B cells pre-incubated with blocking anti-PD-L1/PD-L2 mAbs at 2 weeks after inoculation. Prior to inoculation, blockade of PD-L1/PD-L2 expression was confirmed by reanalysis with FCM (S3 Fig). The SI of anti-Balb/c CD4^+^ and CD8^+^ T cells was significantly higher in mice receiving PerC B cells preincubated with anti-PD-L1 mAb than in mice receiving PerC B cells preincubated with isotype-matched control Abs and in uninoculated control mice (Fig 5). Similar results were obtained for mice receiving PerC B cells preincubated with both anti-PD-L1 and anti-PD-L2 mAbs, but any additive effects of the combination of these mAbs were noted. The SI of anti-Balb/c CD4^+^ and CD8^+^ T cells was the highest in mice receiving PerC B cells preincubated with anti-PD-L2 mAb, although the difference in SIs between mice receiving PerC B cells preincubated with anti-PD-L1 mAb and those receiving PerC B cells preincubated with anti-PD-L2 mAb was not statistically significant. Consistently, the PFs of anti-Balb/c CD4^+^ and CD8^+^ T cells was higher in mice receiving PerC B cells preincubated with anti-PD-L1 and/or anti-PD-L2 mAbs than in mice receiving PerC B cells preincubated with isotype-matched control Abs. This may be because expression of both PD-L1 and PD-L2 on allogeneic PerC B cells is required for inhibiting T cell-responses to the cognate allostimulation.

### Inoculation of allogeneic PerC B cells significantly prolonged the survival of subsequently grafted cognate allogeneic hearts

**Fig 6:**
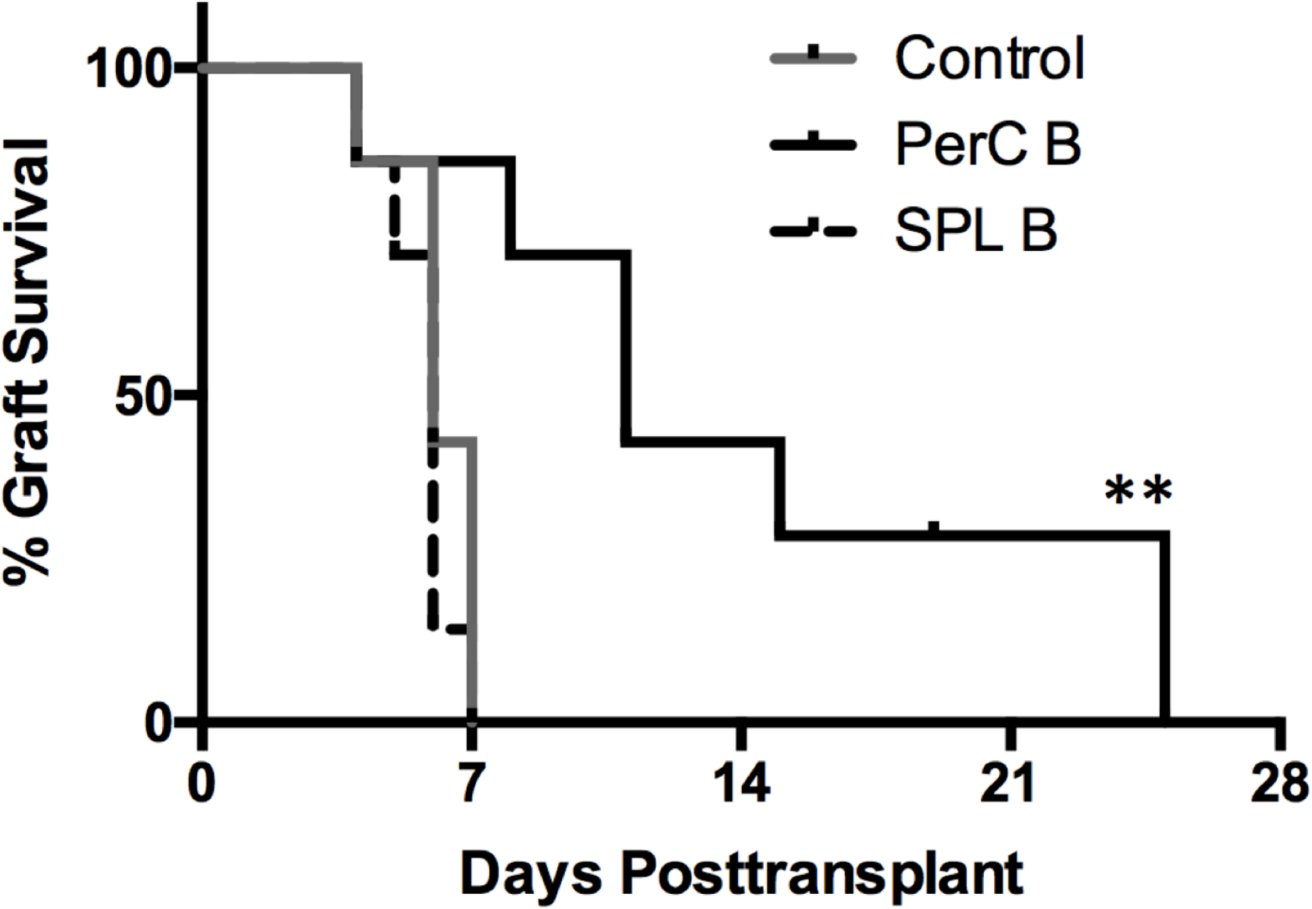
Kaplan-Meier curves of heart allograft survival in mice inoculated with cognate allogeneic SPL or PerC B cells. We investigated the effect allogeneic PerC B cells on prolonging the survival of cognate allografted hearts in a mouse model of heart transplantation. B6 mice were intravenously injected with or without Balb/c SPL or PerC B cells (10 x 10^6^ cells/mouse; n = 7 per group) and were transplanted with the heterotopic hearts of Balb/c mice at 2 weeks after the B cell inoculation. Data are presented as percentage graft survival (**p < 0.01 vs mice in other groups).

To determine whether allogeneic PerC B cells exerted immune-regulatory effects on subsequently transplanted cognate organs, allogeneic hearts of Balb/c mice were transplanted into B6 mice intravenously injected with Balb/c SPL or PerC B cells (10 x 10^6^ cells/mouse) at two weeks injection. Survival curves of the grafted hearts are shown in Fig 6. Untreated B6 mice rejected the hearts of Balb/c within 1-week. B6 mice injected with Balb/c PerC B cells showed significantly prolonged survival of the hearts of Balb/c mice. However, the allografted hearts were eventually rejected within 4-weeks after grafting in these mice. In contrast, B6 mice injected with SPL B cells did not show the prolonged survival of the allografted hearts. Thus, the inoculation of allogeneic PerC B cells could prolong the survival of subsequently grafted cognate allogeneic hearts.

## Discussion

Numerous reports have demonstrated that pretransplant exposure to allogeneic donor antigens through donor leukocyte injection (DLI) or blood transfusion, either alone or in combination with other treatments, can facilitate donor-specific hyporesponsiveness. Some studies have investigated the subsets of allogeneic donor cells that may be important for inhibiting anti-donor immune responses, but the results remain controversial [37–42]. A previous study demonstrated that a single injection of naïve donor SPL B cells prior to transplantation resulted in significantly prolonged donor-specific skin allograft survival compared to that of the infusion of non-B cells in an MHC class I-mismatched model [43], indicating the important role of donor B cells in prolonging the survival of donor-specific skin allografts. However, a significant immune-regulatory effects of injection with SPL B cells has not been demonstrated in a MHC class I/II-mismatched model using a fully allogeneic combination. We demonstrated that intravenous injection of Balb/c SPL B cells in B6 mice resulted in the remarkable production of anti-Balb/c Abs and enhanced T cell responses to Balb/c-allostimulation in B6 recipient mice. In contrast, the injection of Balb/c PerC B cells, which contained B-1a cells co-expressing PD-L1 and PD-L2, in B6 mice lead to the remarkable suppression of anti-Balb/c Abs and the significant inhibition of T cell responses to Balb/c-allostimulation. The experiments using blocking mAbs targeting PD-L1 and PD-L2 suggest that the expression of both PD-L1 and PD-L2 on allogeneic PerC B cells is required to inhibit T cell-responses to the cognate allostimulation through engaging PD-1. Thus, PD-L1^+^ PD-L2^+^ B-1a cells from PerC may be optimal as a subset of allogeneic DLI for inhibiting anti-donor alloimmune-responses.

PD-L1 and PD-L2 coexpression on B-1a cells potentially has important regulatory functions in alloimmune reponses. Since PD-L1^+^ PD-L2^+^ B-1a cells share most of the surface markers with PD-L1^+^ PD-L2^-^ B-1a cells, it is very difficult to sort PD-L2^+^ B-1a cells without using anti-PD-L2 mAb, which blocks and interferes with PD-L2 physiological function. To avoid this problem, an endogenous indicator system for tracking and sorting PD-L1^+^ PD-L2^+^ B-1a cells is highly desirable for applying this particular subset of B cells to DLI prior to allografting.

Even without sorting PD-L1^+^ PD-L2^+^ B-1a cells, the injection of unfractionated PerC B cells resulted in the avoidance of allo-sensitization, and even induced T cell-hyporesponsiveness in response to allostimulation. Inoculation of allogeneic PerC B cells significantly prolonged the survival of subsequently grafted cognate allogeneic hearts; however, the heart allografts were eventually rejected. One possible explanation for the limited prolongation of allograft survival by allogeneic PerC B cells is that these cells may exert efficient suppressive effects on T cells with direct allospecificity, but not on T cells with indirect allospecificity, which are also involved in rejecting allografted organs. To address this issue, it is necessary to determine the mechanisms underlying the role of PD-L1 and PD-L2 expressed on allogeneic PerC B cells in inhibiting T cells that directly recognize alloantigens.

In conclusion, the allogeneic PerC B cells, including the MHC class II^+^ CD80^+^ CD86^+^ PD-L1^+^ PD-L2^+^ B-1a cell subset, have the potential to suppress T cells responding to the cognate allostimulation.

## Supporting information

S1 Fig Phenotypic analysis of B cells in each organ of B6 mice. We investigated the phenotypic characteristics of naïve B cells isolated from the PerC, SPL, liver, BM, PB, and CLN of B6 mice performing an anatomical screening of immune-regulatory markers PD-L1, PD-L2, FasL, and TRAIL, and co-stimulatory membrane markers CD80, CD86, and I-A/I-E (MHC class II) by FCM analysis. (A) Histograms indicating representative FCM results of phenotypic analysis of B cells. Dashed lines indicate isotype-matched control IgG. (B) Percentages (mean ± SEM) expression of each membrane marker expressed on B cells from each organ are shown. *p < 0.05, **p < 0.01, ***p < 0.001, and ****p < 0.00001(Student’s *t*-test). Data are representative of three experiments with three mice per group.
(TIFF)

S2 Fig Division of B-cell subsets from SPL, PerC and liver by FCM assay. Naïve B cells from SPL, PerC and liver were stained with various combinations of mAbs directed against IgM, CD19, CD21, CD11b, CD5, PD-L1, PD-L2, FasL, TRAIL, CD80, CD86, and MHC class II, and analyzed by FCM. Then, these cells were divided into subsets: CD21^int^IgM^int^ follicular B cells, CD21^high^IgM^high^ marginal zone B, and CD21^-/low^IgM^high^ B-0 cells in the spleen, and CD11b^+^CD5^+^ B-1a cells, CD11b^+^CD5^-^ B-1b, and CD11b^-^CD5^-^ B-2 cells in PerC and liver.
(TIFF)

S3 Fig PD-L1/PD-L2 expression on B cells pre-incubated with anti-PD-L1/PD-L2 mAb. Anti-PD-L1 mAb and/or anti-PD-L2 mAb was added to isolated PerC B cells with incubation for 30 min at 37°C in a 5% CO_2_ incubator. Isotype-matched control IgG was used instead of anti-PD-L1 or anti-PD-L2 mAb; Rat IgG2b for anti-PD-L1 mAb, Rat IgG2a for anti-PD-L2 mAb. None of isotype-matched control IgGs interfered with the expression of PD-L1 or PD-L2 on PerC B cells. PD-L1 and PD-L2 expression on B cells was analyzed by FCM assay. Anti-CD19 mAb was used as a B cell marker. Dashed lines on histograms represented isotype-matched control.
(TIFF)

## References

[1] Noorchashm H, Reed AJ, Rostami SY, Mozaffari R, Zekavat G, Koeberlein B, et al B cell-mediated antigen presentation is required for the pathogenesis of acute cardiac allograft rejection. Journal of immunology (Baltimore, Md.: 1950); 2006;177(11):7715–22. 10.4049/jimmunol.177.11.7715 17114442

[2] Knight A. B‐cell acquisition of antigen: Sensing the surface. European Journal of Immunology. 2015;45(6):1600–4. doi: 10.1002/eji.201545684 25929718 10.1002/eji.201545684

[3] Barnett L, Simkins H, Barnett B, Korn L, Johnson A, Wherry J, et al B cell antigen presentation in the initiation of follicular helper T cell and germinal center differentiation. Journal of Immunology (Baltimore, Md: 1950). 2014;192(8):3607–17. 10.4049/jimmunol.1301284 PMC4380085 24646739

[4] Molnarfi N, Schulze-Topphoff U, Weber M, Patarroyo J, Prod’homme T, Varrin-Doyer M, et al MHC class II–dependent B cell APC function is required for induction of CNS autoimmunity independent of myelin-specific antibodies. The Journal of Experimental Medicine. 2013;210(13):2921–37. doi: 10.1084/jem.20130699 24323356 10.1084/jem.20130699 PMC3865476

[5] Rosser E, Mauri C. Regulatory B cells: origin, phenotype, and function. Immunity. 2015;42(4):607–12. doi: 10.1016/j.immuni.2015.04.005 25902480 10.1016/j.immuni.2015.04.005

[6] Yanaba K, Bouaziz J-D, Haas K, Poe J, Fujimoto M, Tedder T. A regulatory B cell subset with a unique CD1dhiCD5+ phenotype controls T cell-dependent inflammatory responses. Immunity. 2008;28(5):639–50. doi: 10.1016/j.immuni.2008.03.017 18482568 10.1016/j.immuni.2008.03.017

[7] Evans J, Chavez-Rueda K, Eddaoudi A, Meyer-Bahlburg A, Rawlings D, Ehrenstein M, et al Novel suppressive function of transitional 2 B cells in experimental arthritis. Journal of Immunology (Baltimore, Md: 1950). 2007;178(12):7868–78. 10.4049/jimmunol.178.12.7868 17548625

[8] Chesneau M, Michel L, Degauque N, Brouard S. Regulatory B cells and tolerance in transplantation: from animal models to human. Frontiers in Immunology. 2013;4:497 doi: 10.3389/fimmu.2013.00497 24427159 10.3389/fimmu.2013.00497 PMC3876023

[9] Rosser E, Blair P, Mauri C. Cellular targets of regulatory B cell-mediated suppression. Molecular Immunology. 2014;62(2):296–304. doi: 10.1016/j.molimm.2014.01.014 24556109 10.1016/j.molimm.2014.01.014

[10] Yoshizaki A, Miyagaki T, DiLillo D, Matsushita T, Horikawa M, Kountikov E, et al Regulatory B cells control T-cell autoimmunity through IL-21-dependent cognate interactions. Nature. 2012;491:864–8. 10.1038/nature11501 PMC3493692 23064231

[11] Klinker M, Reed T, Fox D, Lundy S. Interleukin-5 supports the expansion of Fas ligand-expressing killer B cells that induce antigen-specific apoptosis of CD4+ T cells and secrete interleukin-10. PLoS ONE. 2013;8(8):e70131 doi: 10.1371/journal.pone.0070131 23940537 10.1371/journal.pone.0070131 PMC3734024

[12] Ding Q, Yeung M, Camirand G, Zeng Q, Akiba H, Yagita H, et al Regulatory B cells are identified by expression of TIM-1 and can be induced through TIM-1 ligation to promote tolerance in mice. The Journal of Clinical Investigation. 2011;121(9):3645–56. doi: 10.1172/JCI46274 21821911 10.1172/JCI46274 PMC3163958

[13] DiLillo D, Matsushita T, Tedder T. B10 cells and regulatory B cells balance immune responses during inflammation, autoimmunity, and cancer. Annals of the New York Academy of Sciences. 2010;1183(1):38–57. 20146707 10.1111/j.1749-6632.2009.05137.x

[14] Kalampokis I, Yoshizaki A, Tedder T. IL-10-producing regulatory B cells (B10 cells) in autoimmune disease. Arthritis Research & Therapy. 2013;15:S1. 10.1186/ar3907 PMC3624502 23566714

[15] Wang R.X, Yu C.R, Dambuza I, Mahdi R, Dolinska M, Sergeev Y, et al Interleukin-35 induces regulatory B cells that suppress autoimmune disease. Nature Medicine. 2014;20(6):633–41. doi: 10.1038/nm.3554 24743305 10.1038/nm.3554 PMC4048323

[16] Lal G, Nakayama Y, Sethi A, Singh A, Burrell B, Kulkarni N, et al Interleukin-10 from marginal zone precursor B-cell subset is required for costimulatory blockade-induced transplantation tolerance. Transplantation. 2015;99(9):1817–28. doi: 10.1097/TP.0000000000000718 25839706 10.1097/TP.0000000000000718 PMC4551603

[17] Zhong X, Tumang JR, Gao W, Bai C, Rothstein TL. PD-L2 expression extends beyond dendritic cells/macrophages to B1 cells enriched for V(H)11/V(H)12 and phosphatidylcholine binding. Eur J Immunol. 2007;37(9):2405–10. doi: 10.1002/eji.200737461 17683117 10.1002/eji.200737461

[18] Kilkenny C, Browne W, Cuthill I, Emerson M, Altman D. B cells and programmed death-ligand 2 signaling are required for maximal interferon-γ recall response by splenic CD4+ memory T cells of mice vaccinated with mycobacterium tuberculosis Ag85B. PLoS Biology. PLoS Biology; 2010;8(6):e1000412 doi: 10.1371/journal.pbio.1000412 20613859

[19] Wang H, Lin J, Li P, Skinner J, Leonard W, Morse H. New insights into heterogeneity of peritoneal B‐1a cells. Annals of the New York Academy of Sciences. 2015;1362:68–76 doi: 10.1111/nyas.12791 25988856 10.1111/nyas.12791 PMC4651667

[20] Khan A, Hams E, Floudas A, Sparwasser T, Weaver C, Fallon P. PD-L1hi B cells are critical regulators of humoral immunity. Nat Commun. nature; 2015;6:5997 doi: 10.1038/ncomms6997 25609381 10.1038/ncomms6997

[21] Rozali E, Hato S, Robinson B, Lake R, Lesterhuis J. Programmed death ligand 2 in cancer-induced immune suppression. Clinical & Development Immunology; 2012;2012:656340. 10.1155/2012/656340 PMC3350956 22611421

[22] Tanaka Y, Ohdan H, Onoe T, Asahara T. Multiparameter flow cytometric approach for simultaneous evaluation of proliferation and cytokine-secreting activity in T cells responding to allo-stimulation. Immunological Investigations. 2004;33(3):309–24. 15495790 10.1081/imm-120038079

[23] Onoe T, Ohdan H, Tokita D, Shishida M, Tanaka Y, Hara H, et al Liver sinusoidal endothelial cells tolerize T cells across MHC barriers in mice. Journal of Immunology. 2005;175(1):139–46. 10.4049/jimmunol.175.1.139 15972640

[24] Ohdan H, Yang Y-G, Shimizu A, Swenson K, Sykes M. Mixed chimerism induced without lethal conditioning prevents T cell–and anti-Galα1,3Gal–mediated graft rejection. The Journal of Clinical Investigation. 1999;104(3):281–90. doi: 10.1172/JCI6656 10430609 10.1172/JCI6656 PMC408419

[25] Oliver A, Martin F, Gartland L, Carter R, Kearney J. Marginal zone B cells exhibit unique activation, proliferative and immunoglobulin secretory responses. European Journal of Immunology. 1997;27(9):2366–74. doi: 10.1002/eji.1830270935 9341782 10.1002/eji.1830270935

[26] Kraal G. Cells in the marginal zone of the spleen. Internaitonal Review of Cytology. 1992;132:31–74. 10.1016/s0074-7696(08)62453-5 1555921

[27] Kantor A, Stall A, Adams S, Herzenberg L, Herzenberg L. Differential development of progenitor activity for three B-cell lineages. Proceedings of the National Academy of Scieces. 1992;89(8):3320–4. 10.1073/pnas.89.8.3320 PMC48858 1565622

[28] Hastings W, Tumang J, Behrens T, Rothstein T. Peritoneal B‐2 cells comprise a distinct B‐2 cell population with B‐1b‐like characteristics. European Journal of Immunology. 2006;36(5):1114–23. doi: 10.1002/eji.200535142 16609926 10.1002/eji.200535142

[29] Montecino-Rodriguez E, Dorshkind K. B-1 B cell development in the fetus and adult. Immunity. 2012;36(1):13–21. doi: 10.1016/j.immuni.2011.11.017 22284417 10.1016/j.immuni.2011.11.017 PMC3269035

[30] Zhou Ohdan, Asahara. Calcineurin inhibitors block B-1 cell differentiation: the relevance to immunosuppressive treatment in ABO-incompatible transplantation. Transplantation Proceedings. 2005;37(4):1808–11. doi: 10.1016/j.transproceed.2005.03.129 15919474 10.1016/j.transproceed.2005.03.129

[31] Tazawa H, Irei T, Tanaka Y, Igarashi Y, Tashiro H, Ohdan H. Blockade of invariant TCR-CD1d interaction specifically inhibits antibody production against blood group A carbohydrates. Blood. 2013;122(15):2582–90. doi: 10.1182/blood-2012-02-407452 23943651 10.1182/blood-2012-02-407452 PMC3795459

[32] Montecino-Rodriguez E, Leathers H, Dorshkind K. Identification of a B-1 B cell–specified progenitor. Nature Immunology. 2006;7(3):293–301. doi: 10.1038/ni1301 16429139 10.1038/ni1301

[33] Ghosn E, Yang Y, Tung J, Herzenberg L, Herzenberg L. CD11b expression distinguishes sequential stages of peritoneal B-1 development. Proceedings of the National Academy of Sciences. 2008;105(13):5195–200. 10.1073/pnas.0712350105 PMC2278228 18375763

[34] Wu D, Takahashi K, Murakami K, Tani K, Koguchi A, Asahina M, et al B-1a, B-1b and conventional B cell lymphoma from enzootic bovine leukosis. Veterinary Immunology and Immunopathology. 1996;55(1–3):63–72. 9014306 10.1016/s0165-2427(96)05631-0

[35] Choi Y, Baumgarth N. Dual role for B-1a cells in immunity to influenza virus infection. The Journal of Experimental Medicine. 2008;205(13):3053–64. doi: 10.1084/jem.20080979 19075288 10.1084/jem.20080979 PMC2605232

[36] Muzzio D, Soldati R, Rolle L, Zygmunt M, Zenclussen A, Jensen F. B-1a B cells regulate T cell differentiation associated with pregnancy disturbances. Frontiers in Immunology. 2014;5:6 doi: 10.3389/fimmu.2014.00006 24478775 10.3389/fimmu.2014.00006 PMC3896948

[37] Chai J, Ratnasothy K, Bucy P, Noelle R, Lechler R, Lombardi G. Allospecific CD4+ T cells retain effector function and are actively regulated by Treg cells in the context of transplantation tolerance. European Journal of Immunology. 2015;45(7):2017–27. doi: 10.1002/eji.201545455 25944401 10.1002/eji.201545455

[38] Sandner SE, Clarkson MR, Salama AD, Sanchez-Fueyo A, Yagita H, Turka LA, et al Mechanisms of tolerance induced by donor-specific transfusion and ICOS-B7h blockade in a model of CD4+ T-cell-mediated allograft rejection. American Journal of Transplantation. 2005;5(1):31–9. doi: 10.1111/j.1600-6143.2004.00640.x 15636609 10.1111/j.1600-6143.2004.00640.x

[39] Du J-F, Wang, Wang W-Z, Li M-B, Liang H-L, Guan W-X. Transferable cardiac allograft acceptance induced by transfusion of donor B cells with impaired inducible costimulator/B7h allorecognition. Transplantation Proceedings. 2009;41(5):1840–3. doi: 10.1016/j.transproceed.2009.01.106 19545740 10.1016/j.transproceed.2009.01.106

[40] Marti H, Henschkowski J, Laux G, Vogt B, Seiler C, Opelz G, et al Effect of donor‐specific transfusions on the outcome of renal allografts in the cyclosporine era. Transplantation International. 2006;19(1):19–26. 10.1111/j.1432-2277.2005.00233.x 16359373

[41] Young K, Yang L, Phillips J, Zhang L. Donor-lymphocyte infusion induces transplantation tolerance by activating systemic and graft-infiltrating double-negative regulatory T cells. Blood. 2002;100(9):3408–14. doi: 10.1182/blood-2002-01-0235 12384444 10.1182/blood-2002-01-0235

[42] Brennan DC, Mohanakumar T, Flye MW. Donor-specific transfusion and donor bone marrow infusion in renal transplantation tolerance: a review of efficacy and mechanism. American Journal of Kidney Diseases; 1995;26(5):701–15. 7485121 10.1016/0272-6386(95)90432-8

[43] Gao J, McIntyre M, D’Souza C, Zhang L. Pretransplant infusion of donor B cells enhances donor-specific skin allograft survival. Plos One. 2013;8(10):e77761 doi: 10.1371/journal.pone.0077761 24204953 10.1371/journal.pone.0077761 PMC3810130

