# Supplementary figures and images for: PD-L1/PD-L2-expressing B-1 cells inhibit alloreactive T cells in mice
Source: PLoS One. 2017 Jun 1;12(6):e0178765. doi: 10.1371/journal.pone.0178765 (PMC5453578; doi:10.1371/journal.pone.0178765)

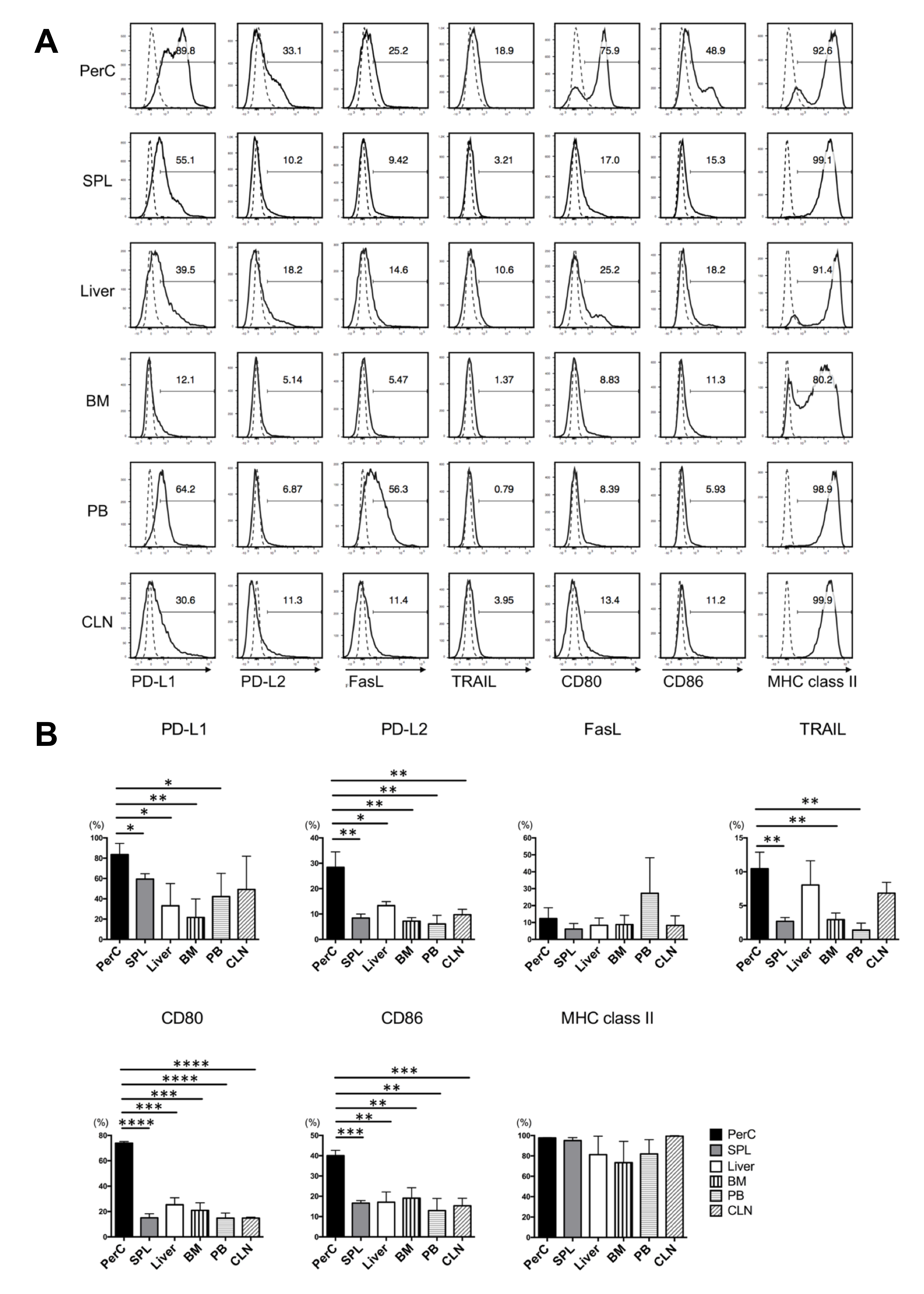

Supplement: S1 Fig — We investigated the phenotypic characteristics of naïve B cells isolated from the PerC, SPL, liver, BM, PB, and CLN of B6 mice performing an anatomical screening of immune-regulatory markers PD-L1, PD-L2, FasL, and TRAIL, and co-stimulatory membrane markers CD80, CD86, and I-A/I-E (MHC class II) by FCM analysis. (A) Histograms indicating representative FCM results of phenotypic analysis of B cells. Dashed lines indicate isotype-matched control IgG. (B) Percentages (mean ± SEM) expression of each membrane marker expressed on B cells from each organ are shown. *p < 0.05, **p < 0.01, ***p < 0.001, and ****p < 0.00001(Student’s t-test). Data are representative of three experiments with three mice per group. (TIFF) [file pone.0178765.s001.tiff]

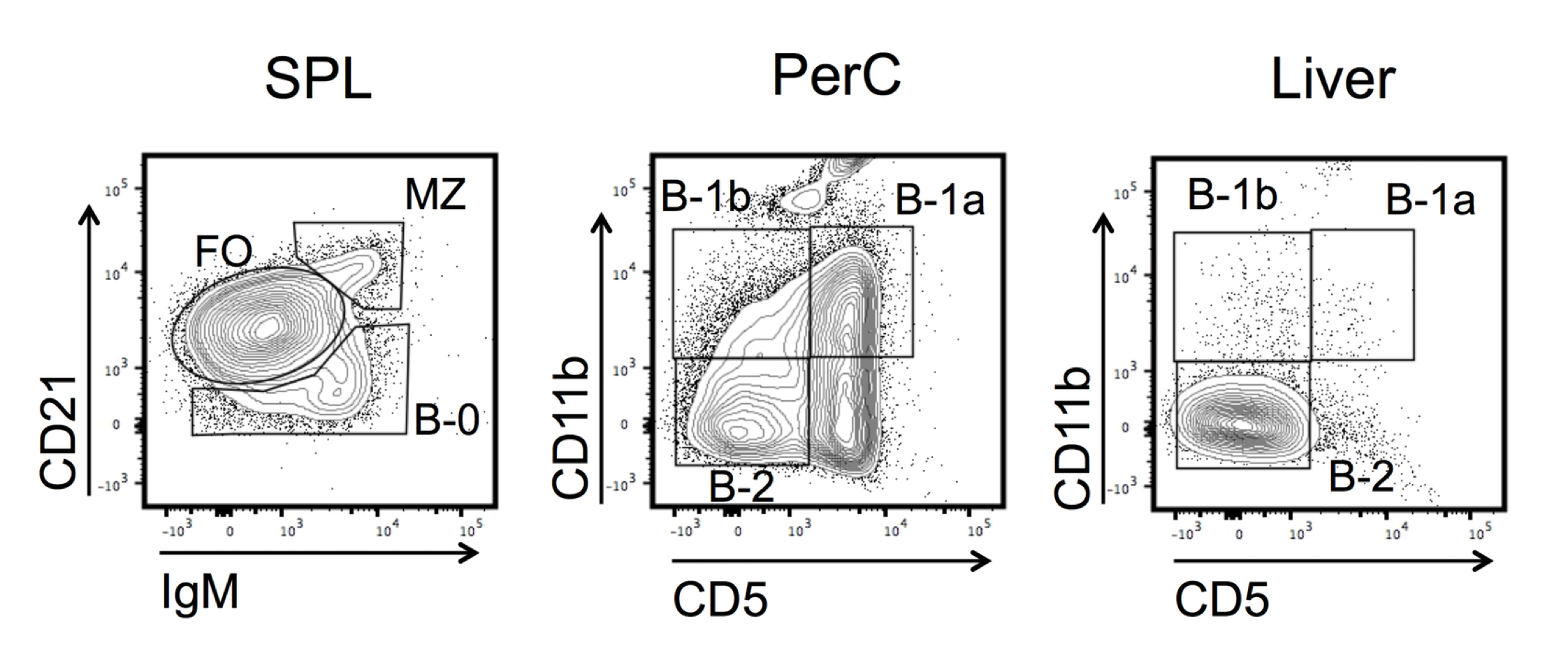

Supplement: S2 Fig — Naïve B cells from SPL, PerC and liver were stained with various combinations of mAbs directed against IgM, CD19, CD21, CD11b, CD5, PD-L1, PD-L2, FasL, TRAIL, CD80, CD86, and MHC class II, and analyzed by FCM. Then, these cells were divided into subsets: CD21intIgMint follicular B cells, CD21highIgMhigh marginal zone B, and CD21-/lowIgMhigh B-0 cells in the spleen, and CD11b+CD5+ B-1a cells, CD11b+CD5- B-1b, and CD11b-CD5- B-2 cells in PerC and liver. (TIFF) [file pone.0178765.s002.tiff]

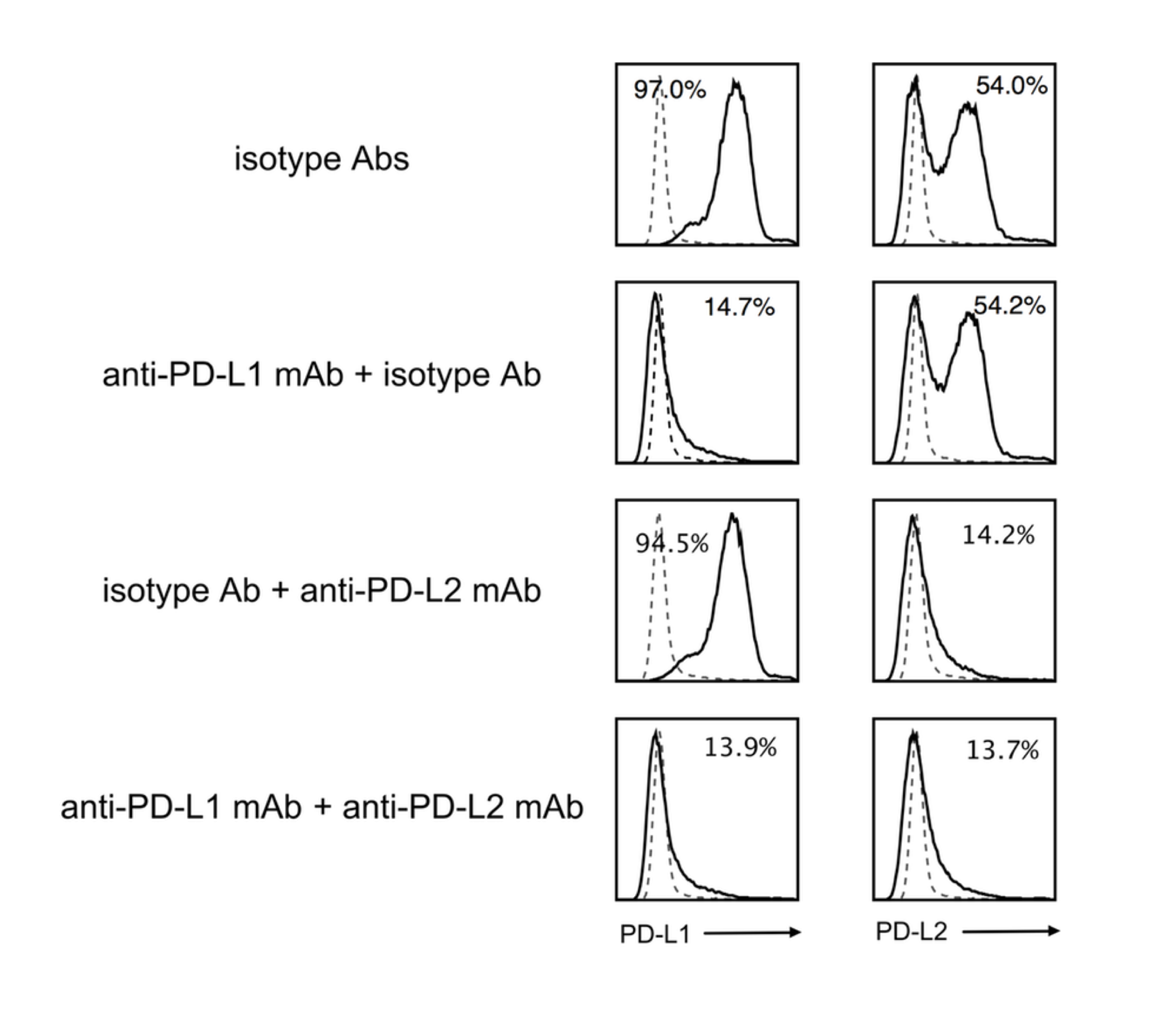

Supplement: S3 Fig — Anti-PD-L1 mAb and/or anti-PD-L2 mAb was added to isolated PerC B cells with incubation for 30 min at 37°C in a 5% CO2 incubator. Isotype-matched control IgG was used instead of anti-PD-L1 or anti-PD-L2 mAb; Rat IgG2b for anti-PD-L1 mAb, Rat IgG2a for anti-PD-L2 mAb. None of isotype-matched control IgGs interfered with the expression of PD-L1 or PD-L2 on PerC B cells. PD-L1 and PD-L2 expression on B cells was analyzed by FCM assay. Anti-CD19 mAb was used as a B cell marker. Dashed lines on histograms represented isotype-matched control. (TIFF) [file pone.0178765.s003.tiff]
